# Perioperative medical management for patients with RA, SPA, and SLE undergoing total hip and total knee replacement: a narrative review

**DOI:** 10.1186/s41927-018-0008-9

**Published:** 2018-01-30

**Authors:** Susan M. Goodman, Anne R. Bass

**Affiliations:** Department of Medicine, Weill Cornell Medical School, Division of Rheumatology Hospital for Special Surgery, 535 E 70th St, New York City, NY 10021 USA

**Keywords:** Total hip arthroplasty, Total knee arthroplasty, Rheumatoid arthritis, Systemic lupus erythematosus, Spondyloarthritis, Perioperative infection, Cardiac risk, Venous-thromboembolism

## Abstract

Total hip (THA) and total knee arthroplasty (TKA) are widely used, successful procedures for symptomatic end stage arthritis of the hips or knees, but patients with rheumatoid arthritis (RA), systemic lupus erythematosus (SLE), and spondyloarthritis (SPA) including ankylosing spondylitis (AS) and psoriatic arthritis (PSA) are at higher risk for adverse events after surgery. Utilization rates of THA and TKA remain high for patients with RA, and rates of arthroplasty have increased for patients with SLE and SPA. However, complications such as infection are increased for patients with SLE, RA, and SPA, most of whom are receiving potent immunosuppressant medications and glucocorticoids at the time of surgery. Patients with SLE and AS are also at increased risk for perioperative cardiac and venous thromboembolism (VTE), while RA patients do not have an increase in perioperative cardiac or VTE risk, despite an overall increase in VTE and cardiac disease. This narrative review will discuss the areas of heightened risk for patients with RA, SLE, and SPA, and the perioperative management strategies currently used to minimize the risks.

## Background

Total hip (THA) and total knee arthroplasty (TKA) remain valuable options to relieve pain and improve function caused by end-stage arthritis of the hip and knee, including for patients with rheumatoid arthritis (RA), Spondyloarthritis (SPA) including ankylosing spondylitis(AS) and psoriatic arthritis (PSA) and systemic lupus erythematosus (SLE). While rates of THA and TKA for patients with osteoarthritis have increased markedly over the past decade, projections suggest even further increases in utilization by 2015 [1, 2]. Large joint arthroplasty rates for patients with rheumatoid arthritis (RA) have remained stable while rates of arthroplasty for patients with spondyloarthritis (SPA) have increased by 50%, and rates have doubled for patients with systemic lupus erythematosus (SLE) [3–6]. The increased utilization of potent disease modifying anti-rheumatic drugs (DMARDs) and biologics like the tumor necrosis factor inhibitors (TNFi) has had a clear impact on quality of life for patients with RA, SPA, and SLE, medication use has not been shown to decrease the incidence of large joint arthroplasty for RA, and most RA, PSA, and SLE patients are receiving these immunosuppressant medications at the time of surgery [6–8]. While improvements in pain and function outcomes measured pre and post-operatively are excellent after THA and TKA for patients with RA, SPA, and SLE, adverse events, in particular infection, are increased [9–17]. Ninety day readmission, most commonly for infection, is also increased for patients with RA [18]. Other adverse events increased in patients with SLE include deep venous thrombosis (DVT) and pulmonary embolism (PE), and major acute coronary events (MACE), although patients with RA do not have an increased risk for VTE or MACE after arthroplasty [19, 20]. While the recent literature addressing adverse events in patients with SPA is sparse, increased inpatient complications including VTE and cardiac complications have been described using the Agency for Healthcare Research and Quality National Inpatient Sample, a large publically available inpatient database [21], while older studies have demonstrated an increase in infection for patients with PSA undergoing arthroplasty, but have not been repeated since the use of prophylactic perioperative antibiotics became widespread [22, 23]. For patients with RA, the RA specific experience of the surgeon or surgical team decreases the risk of adverse surgical events, but the volume-outcome relationship with SLE or SPA specific volume has not been described [24]. This review will discuss the increased infectious, cardiac, and thromboembolic adverse events seen after THA and TKA in patients with RA, SPA, and SLE, and the perioperative medical evaluation and management options to decrease risk.

### Infection

Infection is the most common cause for prosthetic joint failure, and the overall risk of infection is increased in patients with RA, SPA, and SLE [11, 25–29]. The age standardized rate of TKA infection was 1.26% for recipients with RA, compared to 0.84% for recipients with OA, with an adjusted Hazard Ratio (HR) of 1.47, *P* = 0.03, confirmed in a recent meta-analysis demonstrating a relative risk of 1.7 for patients with RA [11, 29]. For patients with SLE whose disease was severe enough to warrant hospitalization within 6 months of surgery, a large study based on Taiwan’s National Health Insurance Research Database found the risk of septicaemia to be markedly increased after surgeries including orthopedic procedures (OR = 3.43, 95% CI 2.48 to 4.74) [14]. Immunosuppressant medications including biologics and DMARDs used to treat RA, SPA, and SLE are recognized to increase the risk of infection [30]. Nonetheless, the majority of patients with RA, SPA, and SLE are taking DMARDs, biologics, or other potent immunosuppressant medications at the time of surgery [10, 16, 31, 32]. Medication management, including decisions to continue or withhold medication, is inconsistent, even at high volume centers with experience in arthroplasty for patients with rheumatic diseases [33]. While the increased risk of infection has been attributed to therapy with immunosuppressant medications including DMARDs and biologics, an association with post-operative infection has never been demonstrated directly in randomized controlled trials performed at the time of surgery. The relationship of post-operative infection to anti-rheumatic medication use is unproven, although [32–34] a meta-analysis has demonstrated an increased risk of infection associated with TNFi exposure around the time of surgery when data was pooled [35], a conclusion supported by additional observational studies [15, 34–36]. However, a recent study that used billing data to specify the timing of infliximab infusions in relation to surgery found no clear increase in risk of infection when infliximab was infused within 4 weeks of surgery compared to longer periods of drug withholding [34]. Disease activity and severity may contribute to the risk of peri-operative infection for RA, SPA, and SLE, and might confound the reported association with medication use [14, 37]. Use of glucocorticoids, however, has been consistently associated with an increased risk of infection in multiple settings- including surgery -at dosages above 15 mg./day, yet supraphysiologic doses of glucocorticoids (“stress dose steroids”) are routinely administered on the day of surgery out of concern for hemodynamic instability [38, 39]. Synthetic DMARDs including methotrexate, leflunomide, and sulfasalazine have not been demonstrated to increase the risk of perioperative infection, in a randomized controlled trial of perioperative methotrexate use [40].

Using a consensus based process, after analysis of an extensive literature review, The American College of Rheumatology (ACR) and The American Association of Hip and Knee Surgeons (AAHKS) have collaborated on a recently published guideline for the peri-operative management of anti-rheumatic therapy for patients with rheumatic diseases including RA, SPA, and SLE undergoing THA and TKA [41]. The recommendations weighed the risk of infection versus the risk of disease flare when medications were withheld, and were informed by the input of a patient panel that placed far greater importance on the risk of infection, concurring with the panel of experts [42]. The collaborators recommend continuing DMARDs, withholding biologics, based on the dose interval, and withholding tofacitinib for 7 days prior to surgery. Any withheld medications can be re-started after 2 weeks, if there is no evidence of infection either at the surgical site, or elsewhere, and the wound demonstrates good healing. The panel recommends administering the usual daily dose of glucocorticoid (after careful taper when possible to <20 mg. prednisone) rather than supra-physiologic “stress dose steroids” on the day of THA or TKA, specifically for adults receiving glucocorticoids for treatment of their rheumatic condition, excluding patients with juvenile arthritis who may have received glucocorticoids during development or those patients receiving glucocorticoids for adrenal or pituitary insufficiency from this recommendation (Table 1) [41].

**Table 1:** Medicatons included in this guideline^a^

DMARDs: CONTINUE these medications through surgery.	Dosing Interval	Continue/Withhold
Methotrexate	Weekly	Continue
Sulfasalazine	Once or twice daily	Continue
Hydroxychloroquine	Once or twice daily	Continue
Leflunomide (Arava)	Daily	Continue
Doxycycline	Daily	Continue
BIOLOGICS: STOP these medications prior to surgery and schedule surgery at the end of the dosing cycle. RESUME medications at minimum 14 days after surgery in the absence wound healing problems, surgical site infection or systemic infection.	Dosing Interval	Schedule Surgery (relative to last biologic dose administered)
Adalimumab (Humira) 40 mg	Every 2 weeks	Week 3
Etanercept (Enbrel) 50 mg or 25 mg	Weekly or twice weekly	Week 2
Golimumab (Simponi)50 mg	Every 4 weeks (SQ)or	Week 5
	Every 8 weeks (IV)	Week 9
Infliximab (Remicade)3 mg/kg	Every 4, 6, or 8 weeks	Week 5, 7, or 9
Abatacept (Orencia) weight-based 500 mg; IV 1000 mg; SQ	Monthly (IV)or	Week 5
125 mg	Weekly (SQ)	Week 2
Rituximab (Rituxan)1000 mg	2 doses 2 weeks apart every 4–6 months	Month 7
Tocilizumab (Actemra) IV 4 mg/kg;	Every week (SQ) or Every 4 weeks	Week 3
SQ 162 mg	(IV)	Week 5
Anakinra (Kineret) SQ 100 mg	Daily	Day 2
Secukinumab (Cosentyx) 150 mg	Every 4 weeks	Week 5
Ustekinumab (Stela) 45 mg	Every 12 weeks	Week 13
Belimumab (Benlysta) 10 mg/kg	Every 4 weeks	Week 5
Tofacitinib (Xeljanz) 5 mg: STOP this medication 7 days prior to surgery.	Daily or twice daily	7 days after last dose
SEVERE SLE-SPECIFIC MEDICATIONS: CONTINUE these medications in the perioperative period.	Dosing Interval	Continue/Withhold
Mycophenolate	Twice daily	Continue
Azathioprine	Daily or twice daily	Continue
Cyclosporine	Twice daily	Continue
Tacrolimus	Twice daily (IV and PO)	Continue
NOT-SEVERE SLE: DISCONTINUE these medications in the perioperative period.	Dosing Interval	Continue/Withhold
Mycophenolate	Twice daily	Withhold
Azathioprine	Daily or twice daily	Withhold
Cyclosporine	Twice daily	Withhold
Tacrolimus	Twice daily (IV and PO)	Continue

Dosing intervals obtained from prescribing information provided online by pharmaceutical companies

^a^2016 American College of Rheumatology/American Association of Hip and Knee Surgeons Guidelines for the Perioperative Management of Anti-rheumatic Medication in Patients with Rheumatic Diseases Undergoing Elective Total Hip or Total Knee Arthroplasty. Permission to reuse given by John Wiley and Sons on August 28, 2017, License Number 4177680712679

### Major acute cardiac events

Patients with SLE, SPA, and RA have significantly increased risk of cardiac disease compared to age and sex matched controls. In studies using carotid atherosclerosis as a surrogate for coronary artery disease, patients with RA and SLE had almost 3 times more carotid atherosclerosis (RA 44% versus 15%, *p* = .001; SLE 37.1% vs. 15.2%, *P* < 0.001), even after controlling for traditional cardiac risk factors [43, 44]. For patients with RA, the risk of MI is similar to that seen in patients with diabetes [19, 45, 46]. While cardiac mortality is increased by 50% for patients with RA, they are less likely to report cardiac symptoms such as chest pain [47, 48]. Traditional risk factors such as hypertension and smoking contribute to cardiac risk for patients with RA, and markers of sustained inflammation and disease severity are additional risk factors [49–51]. For patients with SLE, mortality from cardiovascular disease has continued to increase, despite improvements in mortality previously seen for patients with active SLE. The risk of death and cardiac events has doubled, with increases seen even early in the disease [52, 53]. Young women with SLE between the age of 35–44 are 50 times more likely to have an MI than age matched controls [54]. While the extreme increase in relative risk for cardiovascular events is dramatic in young patients with SLE, the absolute risk of cardiovascular events is higher in older women with SLE [55]. In a multicenter Spanish SLE register, 374 (10.9%) of patients had angina, an MI, stroke, or peripheral artery disease. In this cohort, traditional risk factors included smoking and hyperlipidemia, but hypocomplementemia was a risk factor as well, suggesting that for SLE, disease activity contributes to cardiovascular risk [56]. Others have also found that traditional risk factors alone cannot explain the increased risk, and factors such as SLE disease activity and severity contribute to cardiovascular risk [55–58].

Patients with SPA have a higher prevalence of CVD, and have a significant increase in traditional CVD risk factors including hypertension, hyperlipidemia as well as obesity [59–61]. For patients with PSA, the risk of major cardiac events is increased for those not prescribed a DMARD (HR 1.24, 95% CI 1.03 to 1.49), suggesting that either disease severity or activity may also increase cardiac risk in PSA [62].

Perioperative cardiovascular risk is increased in those with known cardiovascular disease. In a large orthopedic hospital, post-operative myocardial infarction (MI) occurred in 0.6% of 8000 inpatient orthopedic procedures, while the risk for those with known ischemic heart disease or risk factors for ischemic heart disease increased to 6.5% [63] suggesting that patients with RA, SPA, and SLE should be at higher risk, given the increase in the prevalence of cardiac disease for these patients and the similarity in risk profile for RA patients when compared to patients with diabetes [45, 54, 57, 64, 65] . However, when a large insurance data-base was queried and patients with RA were compared to patients with DM after surgery, there was a substantially lower rate of cardiac events (RA 0.34% vs. DM 1.07%; *p* < 0.001) and death (RA 0.30% vs. DM 0.65%; *p* < 0.001) for patients with RA after intermediate risk procedures including arthroplasty [19]. In addition, there was no increase in in-hospital mortality for RA patients after arthroplasty when compared to controls [66]. However, for patients with SLE, unlike in patients with RA, perioperative risk of cardiac events and death were significantly increased in the US Nationwide Inpatient Sample, with an OR of 4.0 (95% CI 1.9–8.0) for postoperative mortality with hip replacements and an OR of 1.2 (95% CI 0.2–7.5) for mortality with knee replacements [20, 66]. Increased in-hospital mortality for patients with SLE has been confirmed using discharge data from seven states, comprising 8 million discharges, with a higher risk of in-hospital mortality(OR (99% CI) of 1.27 (1.11, 1.47); *P* < .001), although no increase in in-hospital cardiac events were reported [67]. Similarly, 30 day post-operative mortality risk is increased in patients with SLE in a report using the Taiwan national insurance database (OR = 2.39, 95% CI 1.28 to 4.45) [14]. For patients with AS, the risk of in-patient cardiac events after THA was significantly higher than in controls [21].

The American Heart Association and American College of Cardiology (AHA/ACC) have formulated guidelines for assessing cardiac risk in preparation for surgery, based on a combination of functional capacity and risk factors including the presence of CAD (angina and/or prior MI), heart failure, stroke or transient ischemic attack, renal insufficiency (serum creatinine [2 mg/dl or creatinine clearance \60 ml/min/1.73 m2), and diabetes requiring insulin therapy [68]. Using this guideline in patients with RA, SPA, and SLE is complicated by the poor functional capacity of many patients prior to THA and TKA, when poor functional capacity is defined as the inability to achieve at least 4 Metabolic Equivalents (METS), achieved by light shoveling, dancing, or gardening, defining “light” as when the activity results in “only minimal perspiration and only a slight increase in breathing above normal” [69]. Moreover, patients with RA with cardiovascular disease may not have symptoms [48, 70, 71]. Using the classic Framingham risk equation (based on age, sex, total cholesterol level, high density lipoprotein cholesterol level, smoking history, and systolic blood pressure), patients with RA, SPA, and SLE may fall into a low risk category, leading some to add the presence of a systemic inflammatory disease such as RA, SPA, and SLE to the list of traditional cardiovascular risk factors, or to add a multiplication factor of 1.4 to the calculation of cardiac risk, recognizing that the current risk assessment tools are unreliable and underestimate cardiac risk in patients with RA, SPA, and SLE [51, 72–76]. Major orthopedic surgery is categorized as an intermediate risk procedure in the ACA/AHA guideline, and carries a 1–5% risk of MI or cardiovascular death [68]. While none of these formulations are entirely satisfactory for estimating perioperative risk in patients with inflammatory diseases, a pragmatic approach has been to include RA, SPA, and SLE as risk factors in the ACA/AHA algorithm; patients with 2 risk factors, one of which could be RA, SPA, and SLE, and poor functional capacity (< 4 METS), would undergo testing for evidence of cardiac ischemia prior to elective intermediate risk surgery.

### Venous Thromboembolism

Patients with rheumatic diseases have more than double the risk of VTE compared to the general population, particularly when their disease is active [77–81]. This is not surprising given the association between inflammation and thrombosis [82]. Although RA patients have double the risk of VTE over all [83–85], RA patients undergoing arthroplasty are not at increased VTE risk [11, 32, 85, 86]. For example, in a study of close to a billion hospitalized patients, RA and non-RA patients admitted for surgery on their joints had the same risk of postoperative VTE (0.67%) [85]. In contrast, the RA patients admitted for other reasons had double the risk of VTE compared to their non-RA peers (2.3% vs. 1.15%) [85]. Risk factors for postoperative VTE in RA patients in this study were similar to those in other patients and included advanced age, female gender, and African–American race. It is possible that good disease control in RA patients undergoing arthroplasty explains their relatively low VTE risk. In contrast, a recent retrospective study of patients undergoing spine surgery demonstrated a significantly higher risk of VTE in RA patients than non-RA patients [87]. Although this finding requires validation, it could also reflect the more urgent nature of spine surgery, which may not permit RA disease optimization.

Patients with SPA are at higher risk of VTE than the general population [79]. As with RA patients, however, the risk of VTE after arthroplasty does not appear to be higher in these patients. For example, in a study that looked at complication rates after TKA, there was a higher rate of DVT, but no difference in the rate of PE (or total VTE) in 4575 ankylosing spondylitis patients, and no difference in DVT or PE rates in the 7918 psoriatic arthritis compared to 1,751,938 OA controls [88].

Patients with SLE are also at higher risk for VTE [79–81, 89, 90]. Among lupus patients, VTE risk factors include smoking, obesity, hemolytic anemia and anti-phospholipid antibodies, while Asian race is protective [89, 90]. Lupus patients who have received inpatient care for their lupus in the 6 months prior to surgery are five times more likely to experience postoperative PE [14] reinforcing the concept that it is disease activity that increases thrombotic risk. Lupus patients are also more likely than other individuals to have antiphospholipid antibodies, a well-established risk factor for VTE [89, 91]. Among patients with antiphospholipid antibodies, those with a lupus anticoagulant (LAC) or triple positivity (LAC plus high titer anti-cardiolipin and anti-beta 2 glycoprotein 1 antibodies) are at highest VTE risk [92–94].

Current recommendations for postoperative VTE prophylaxis in patients with the antiphospholipid antibody syndrome (APS) are to minimize the time off anticoagulation, bridge with low molecular weight heparin, and resume warfarin the night of surgery [95, 96]. Although a recent prospective trial demonstrated less thrombin generation in APS patients given rivaroxaban compared to warfarin [97], there are no studies demonstrating the safety and/or efficacy of direct oral anticoagulants in preventing thrombotic events in APS. In patients with APS, surgery can sometimes act as a trigger for catastrophic antiphospholipid syndrome (CAPS). Patients with CAPS should be managed with parenteral anticoagulation and may require additional treatment with corticosteroids, IVIG and/or rituximab [98] .

It should also be remembered that, in addition to assessing the activity of their rheumatic disease, patients with RA, SPA and SLE should also be assessed for traditional VTE risk factors prior to surgery (Table 2).

**Table 2:** Risk factors for venous thromboembolism in rheumatic disease patients undergoing surgery

Patient-specific risk factors	
Active rheumatic disease	
History of VTE^a^	
Active cancer	
Estrogen	
Active cancer	
Smoking	
Advanced age	
Black race (Asian race protective)	
Obesity	
Non-O ABO blood group	
Thrombophilia	
Surgery-specific risk factors	
Orthopedic surgery > other surgery	
Hip fracture	
Bilateral arthroplasty	
Revision arthroplasty	
General > Axial/regional anesthesia	

^a^VTE = venous thromboembolism

Recommended prophylactic anticoagulation for (non-APS) rheumatic disease patients whose disease is quiet is the same as for non-rheumatic disease patients. Patients with active rheumatic disease should, preferably, have their disease controlled prior to undergoing elective orthopedic surgery. Patients with active rheumatic disease who must undergo urgent surgical procedures should be considered at higher than average VTE risk; prophylaxis will depend on their particular procedure.

## Conclusion

In summary, while patients with RA, SPA, and SLE continue to utilize THA and TKA, they are at higher risk for complications. Risk of infection is higher in patients with RA, SPA, and SLE, and may be decreased via perioperative medication management strategies that include withholding all biologics prior to surgery, although traditional DMARDs do not appear to increase risk of infection. Perioperative cardiac risk stratification is improved with the recognition that patients with RA, SPA, and SLE are at higher risk of cardiac disease than age matched controls. While patients with SLE are at higher risk of VTE after surgery, VTE risk for patients with RA and SPA is no higher than for others undergoing THA and TKA. Improved outcomes may be achieved with attention to pre-operative optimization to minimize perioperative risks.
